# Impact of diet on the immunological microenvironment of the pregnant uterus and its relationship to allergic disease in the offspring – a review of the recent literature

**DOI:** 10.1590/S1516-31802006000500013

**Published:** 2006-09-07

**Authors:** Daniella Campelo Batalha Cox Moore, Pedro Xavier Elsas, Elisabeth Santos Maximiano, Maria Ignez Capella Gaspar Elsas

**Keywords:** Allergy, Pregnancy, Diet, Immunology, Child, Alergia, Gravidez, Dieta, Imunologia, Criança

## Abstract

Medical progress has reduced the mortality from infectious diseases in most countries, but allergic diseases have become more prevalent worldwide over the same period, especially in industrialized countries. This has prompted speculation that modern lifestyles have altered the relationship between heredity and environment so as to promote development of an atopic phenotype when exposure to infection decreases. A healthy uterine microenvironment is known to favor Th2 lymphocyte development. However, some evidence suggests that persistence of the Th2 pattern of immunity directs the developing organism's immune response towards a long-lasting atopic phenotype. Even though the outcome also depends on other factors (such as infection, functional state of the intestinal microflora, and exposure to environmental allergens at times critical to development), it seems that the immune system during the perinatal period is responsive to interventions that are no longer effective in adulthood. We have reviewed the literature accessible through Medline to identify recent advances in the prevention of allergic disease through interventions in the fetal-maternal relationship. Diet seems to have a significant impact on the immunological profile of the pregnant uterus, as well as on the postnatal development of allergic disease in the offspring, as suggested by the effects of probiotic bacteria and by manipulations of the dietary content of polyunsaturated fatty acids and antioxidants. This highlights the need for further studies, in order to define the best intervention methods, the most appropriate time interval and the individuals who will most likely benefit from them.

## RESUMO

Progressos médicos reduziram a mortalidade por doenças infecciosas em muitos países, mas doenças alérgicas tornaram-se mais prevalentes no mundo inteiro, no mesmo período, especialmente nos países industrializados, levando alguns a postular que a vida moderna influencia as relações entre hereditariedade e meio ambiente de forma a favorecer o desenvolvimento de atopia quando a exposição a agentes infecciosos diminui. O micro-ambiente fisiológico do útero gravídico favorece o desenvolvimento de linfócitos Th2. Contudo, a evidência sugere que um padrão persistente de imunidade Th2 direciona a resposta imune do organismo em desenvolvimento para um fenótipo atópico duradouro. Embora o resultado dependa de outros fatores, incluindo infecções, o estado funcional da microflora intestinal, e a exposição a alergenos ambientais em momentos críticos do desenvolvimento, o sistema imune no período perinatal permanece suscetível a intervenções que não têm efeito no adulto. Fizemos uma revisão da literatura acessível através da Medline para identificar avanços recentes na prevenção de doenças alérgicas por meio de intervenção na relação materno-fetal. A dieta parece ter um impacto significativo sobre o perfil imunológico do útero gravídico, assim como sobre o desenvolvimento pós-natal de doença alérgica, como sugerido pelos efeitos de bactérias probióticas e pela manipulação do conteúdo de ácidos graxos poli-insaturados e de antioxidantes na dieta. Isso reforça a necessidade de estudos mais amplos para determinar o melhor tipo de intervenção, o momento mais adequado e os indivíduos que mais serão beneficiados.

## INTRODUCTION

One of the major current concerns in the management of allergies originates in the observation that, even though the worldwide prevalence of asthma (as well as other allergic diseases, including atopic dermatitis) has significantly increased over the last two decades,^1^ studies comparing its prevalence patterns in different countries have failed to pinpoint a specific etiological factor that could account for this increased prevalence.^2^ The morbidity associated with allergic disease, and the high associated costs of medication and/ or inpatient treatment, highlight the need for more studies in order to change this rather unsatisfactory picture.

A number of pathophysiological hypotheses have been advanced, in an attempt to explain this phenomenon, which seems to be reaching its plateau level. Foremost is the so-called hygiene hypothesis, first proposed by Strachan (1989), who had shown that the risks of hay fever and allergic sensitization were inversely proportional to the number of siblings in the families studied. Strachan proposed that the reduction in average family size, the improvement of housing conditions and the shift towards better personal hygiene habits – all trends associated with modern urban life – have contributed towards decreasing the risk of cross-infection between siblings living together. The observation that this apparently improved situation regarding infectious disease is associated with increased risk of allergies suggests that cross-infection has a beneficial effect on the incidence of allergic disease.^3^ These initial epidemiological observations were followed by a number of studies, both clinical and experimental, that attempted to substantiate the hypothesis and to establish a mechanism that could account for the observations. However, at present a number of controversial issues persist, and the hygiene hypothesis is not yet universally accepted.

An alternative view involves the concept of *fetal programming*, or *imprinting*, for which the evidence will be reviewed below. There is growing interest in the possibility that the mother, during pregnancy, may have an important influence on the development of the immune response to environmental allergens in the growing fetus and/or the neonate. The interactions between the maternal organism, the placental microenvironment and the fetus constitute a new frontier for research in allergology and may become a target for therapeutic and/or prophylactic intervention, with the aim of preventing allergic asthma.^4^

## OBJECTIVE

The aim of this review, which is directed towards professionals in the fields of primary healthcare and public health planning/evaluation, was to summarize the current insights on the development of immune reactivity in the fetus, within the context of allergic sensitization during pregnancy, and to outline strategies for reducing the prevalence of allergic disease, based on understanding the mechanisms involved.

## THE LINK BETWEEN SUCCESSFUL PREGNANCY AND A "TH2" MICROENVIRONMENT IN THE PREGNANT UTERUS

There are a few concepts that are prerequisites for any discussion on the immunological aspects of pregnancy. Foremost is the discovery, reported by Mossman et al. in 1986,^5^ that CD4+ T lymphocytes differentiate into two major subpopulations with very distinct functional properties. Their work showed that naïve CD4+ T cells, following their initial stimulation by antigens associated with antigen-presenting cells, would undergo differentiation into cells with distinctive patterns of cytokine production (and hence, distinct regulatory roles). This differentiation would depend on the nature of the initial stimulus, and also on the precise conditions under which stimulation took place. On the basis of their specific cytokine secretion patterns, it was possible to define the Th1 and Th2 subpopulations.

Th1 cells selectively produce interleukin-2, interferon-γ and tumor necrosis factor-γ. These three cytokines are essential for the defense against intracellular microorganisms, including viruses and some bacteria that survive as intracellular parasites, such as *Mycobacterium tuberculosis* and *Listeria monocytogenes*. Interferon-γ plays a special role in activating macrophages, thereby preventing survival and multiplication of microorganisms in their vacuoles and cytoplasm. On the other hand, tumor necrosis factor-γ is required for organizing and maintaining the granulomas that make it possible to contain these microorganisms, and thereby to limit their systemic dissemination. This same set of cytokines is essential for delayed-type hypersensitivity reactions and graft-versus-host reactions, which represent two distinct types of cell-mediated immunity.^6^

In contrast, Th2 cells selectively produce cytokines such as interleukin-4 (important for producing anaphylactic antibodies of immunoglobulin E class, IgE), interleukin-5 (the major growth factor for eosinophils and a critical regulator of antibody production by some B lymphocytes), interleukin-6 (a major mediator of the systemic acute phase reactions, and a nonspecific enhancer of antibody production) and interleukin-13 (also involved in promoting IgE synthesis, as well as in many of the tissue phenomena associated with allergic reactions and asthma). This subset of cytokines is critical for the pathophysiology of acute allergic processes, involving IgE, mast cells and eosinophils, in addition to participating in combating extracellular pathogens, which critically relies on antibody production.^6^

In Mossman's paradigm,^5^ there is a dynamic equilibrium between these two T cell subpopulations: when either one pre-dominates, the other one is correspondingly inhibited. This is broadly demonstrated by the observation that some of the cytokines selectively produced by these subpopulations can influence the development and function of the complementary subpopulation. For instance, interferon-γ (IFN-γ), a Th1 product, inhibits the development and function of Th2 cells by a variety of mechanisms. A large number of studies over the last two decades^7,8^ have shown that an imbalance in the numbers of Th1 and Th2 cells may be at the origin of a great variety of disease processes.

This general paradigm of CD4+ T specialization, however, must be regarded within the context of each specific organ or tissue, because microenvironmental conditions decisively influence the likelihood of initiation and completion of either differentiation program. One such specific setting is pregnancy, which is exclusively found in females and is structured around the need to protect and nurture the developing fetus.

Many clinical and experimental studies have suggested that successful pregnancy takes place in a microenvironment in which the predominant cytokine production pattern is Th2.^9^ In contrast, experimental studies on mice,^10^ and also clinical studies among humans, have shown that a predominantly Th1 cytokine production pattern leads to miscarriages and fetal losses.^11^ It is believed that Th2 cytokines are part of a complex network of immunological interactions that oppose the underlying tendency to reject the fetus. At least in principle, the fetus is endowed with histocompatibility antigens distinct from those found in the maternal organism, and therefore is susceptible to rejection as an allograft. On the other hand, the presence of the same Th2 cytokines promotes an intrauterine microenvironment that is favorable to the progression of allergies. The fetus develops in an environment in which the production of interleukin-4 and the immunosuppressive and anti-inflammatory interleukin-10 predominates over that of interferon-γ.^12^ This, by itself, does not determine the birth of a child prone to allergic disease (and, at any rate, allergic manifestations are rare at birth, even in children who will subsequently become allergic). However, even if babies are not born allergic, there is some evidence that babies who will eventually develop allergic disease show a more pronounced Th2 bias at birth.^13^

A number of other factors may superimpose on the preexisting influences. A study by Brown et al.^14^ showed that the type of delivery may modulate the production of interferon-γ by mononuclear cells isolated from umbilical cord blood. These authors showed that vaginal delivery is associated with high interferon-γ production. In contrast, following delivery by cesarean section, the production of interferon- γ is lower. The authors emphasized the need for further studies to evaluate the duration of these divergent patterns of physiological regulation. However, in any event, their findings indicate that normal delivery is one of the first physiological stimuli to disturb the previously existing Th1-Th2 equilibrium and shift cytokine production from the Th2 pole towards the Th1 pole. Through this, the transition to life in a potentially infectious environment, as opposed to in the highly protected environment of the uterus, is facilitated.

## THE IMPACT OF MATERNAL ALLERGIES ON THE HEALTH OF CHILDREN

The hypothesis that early events in fetal development and in infancy can determine the emergence of allergic disease has arisen from a number of clinical and epidemiological observations. Foremost is the well-established fact that the risk of atopy is higher among the offspring of atopic mothers than among the offspring of atopic fathers. This points towards a strong symbiotic effect between mother and fetus during pregnancy and also between mother and infant after birth.^15^

IgE, the main effector antibody class in the allergic reactions that trigger asthma and allergic rhinitis, is unable to effectively cross the placental barrier. However, the presence of IgE and Th2 cytokines in the amniotic fluid has already been reported.^16^ This means that, in addition to having its skin covered by amniotic fluid containing IgE, the fetus, which aspirates and swallows amniotic fluid, is also exposed to IgE through the lymphoid tissue associated with the respiratory and digestive tracts. If this IgE is complexed with a specific antigen, the minimum conditions for inducing allergen-specific responses in the fetus are fulfilled, since dendritic cells endowed with high-affinity IgE receptors can endocytose the immune complexes. This material can subsequently be processed and presented in the context of Class II major histocompatibility complex (MHC) molecules, thereby leading to activation of CD4+ Tlymphocytes and the emergence of a specific response to these allergens.^17^

A recent study by van Gool et al.^18^ reinforced this hypothesis, by showing that one of the determinants of neonatal serum IgE levels is the presence of elevated maternal serum IgE. The same study showed that the neonatal serum IgE levels were inversely correlated with the order of birth among the offspring of multiparous mothers. This provides an alternative mechanism to account for the "sibling effect" initially detected by Strachan^3^ (1989), as opposed to the previously mentioned hypothesis of protection conferred by cross-infections among the offspring from multiparous mothers.

## ALLERGIC RESPONSIVENESS IN THE FETUS AND NEWBORN

There is considerable speculation in the literature concerning the moment at which the developing human fetus becomes capable of mounting an adaptive immune response. The traditional view is that the newborn's immune system is entirely naïve. However, based on the fact that only immunoglobulin G can cross the placenta in humans, the demonstration of elevated IgE in the cord blood^19^ and the presence of allergen-specific T cells at birth^20^ suggest that the immune system can be stimulated by allergens before birth. It has already been shown that the fetus is capable of mounting antigen-specific responses to rubella antigens and tetanus toxoid. In these studies, immunoglobulin M (IgM) could be detected in cord blood from neonates whose mothers had been infected with rubella or vaccinated against tetanus toxoid during pregnancy.^21,22^ It is important to emphasize that the presence of anti-tetanus toxoid IgM in cord blood is only detected if immunization took place during pregnancy, and not before that. This implies that it is only under this condition that the antigen seems to gain access to the fetal immune system.

It is therefore important to determine whether antigens or allergens are capable of reaching the fetus in the uterine environment. A study on pregnant women who underwent routine amniocentesis around the 16^th^ week of gestation reported the presence of *Derp 1* (the major allergen of *Dermatophagoides pteronissimus*, an important species of house dust mite) and ovalbumin both in amniotic fluid and in maternal blood.^23^ Moreover, that study provided evidence for the existence of two separate pathways for transfer of allergens to the fetus, namely the transplacental and transamniotic routes (even in sterile procedure). A study by Viscarello et al. showed that the p24 antigen of the human immunodeficiency virus (HIV-1) is also detectable in the amniotic fluid and fetal blood of mothers infected by HIV.^24^ The presence of viruses or viral antigens in the maternal serum or amniotic fluid is not synonymous with fetal infection, since the study by Viscarello found two fetal serum samples that were negative for p24 antigen, in spite of its presence in maternal serum and amniotic fluid. Another recent study showed that food allergens can be measured in the maternal circulation and milk soon after ingestion.^25^

## LYMPHOID STRUCTURE MATURATION AND ITS ROLE IN ALLERGEN SENSITIZATION

Several lines of evidence point to the 22^nd^ week of gestation as the critical time at which the immunological reactivity of the fetus becomes detectable.^26^ One of the major points of interest is the fetal gut, which becomes structurally mature around the 19^th^ week, including its defense structures, such as lymphoid follicles, Peyer's patches and M cells.^27^ Hence, the coincidence between the maturation of specific structures and the appearance of immunological competence suggests that the gut development can, at this moment, enable the host to respond to environmental antigens, and may decisively influence what will happen in the postnatal period.

## THE IMPACT OF MATERNAL DIET AND THE EFFECT OF DIETARY INTERVENTION ON ALLERGIES

Over the last two decades, the prevalence of allergic disease has increased in industrialized countries and, in an interesting (and perhaps not coincidental) way, particularly in countries where English is the official language. The mechanisms underlying this increase in allergic phenomena are still not well understood. As the mutation rates are known to be too low to account for any significant change in the genetic profile of the general population over such a short evolutionary time, it is likely that epigenetic factors, i.e. those associated with the environment and modern lifestyles, are involved in this increase in allergic disease. One of the factors suspected of contributing towards this increase is the reduced intake of fresh fruits and vegetables, especially in the UK.^28^ The role this plays in the genesis of allergic disease has not yet been determined; however, it is clear that it contributes towards aggravating preexisting disease.^29^

These observations suggest that dietary interventions may lead to important changes in the likelihood that allergic diseases will develop. Within this context, some of the most interesting observations relate to probiotic effects.

A study by Kalliomaki et al.^30^ highlighted the importance of probiotics as modulators of allergic disease. These researchers carried out a randomized, controlled study in which *Lactobacillus* GG (1 × 10^10^ colony-forming units) was introduced during the prenatal period into the diet of pregnant women who were deemed to present the risk of transmitting atopy (namely, those who had at least one first-degree relative affected by eczema, or a partner with atopic eczema, rhinitis or asthma). Probiotics were also administered to infants during their first six months of life. The study showed that the frequency of atopic eczema at two years of age in the probiotic consumption group was half of what it was in the placebo group. This result shows that *Lactobacillus* GG is effective in preventing atopic eczema.

Probiotics, especially those derived from strains of *Lactobacillus* and *Bifidobacterium*, are capable of modifying the *in vitro* pattern of cytokine production by monocytes and lymphocytes, thereby enhancing the production of interleukin-12 and interferon-γ, two of the hallmarks of the Th1 profile.^31^ This observation points towards one of the possible mechanisms through which probiotics can have such a marked influence on the incidence of childhood atopy. Within this conceptual framework, probiotics can represent a stimulus capable of inducing the fetal immune system, which had hitherto been biased towards the Th2 profile, to find a new equilibrium point without concurrently inflicting damage on the organism, such as would be caused by a viral or bacterial infection, with the accompanying risk to the continuity of gestation.

A study by Pohjavuori et al.^32^ showed that probiotic bacteria are also potentially beneficial for the maturation of the immune system in the infant. These investigators carried out a double-blind randomized study in which patients with suspected allergy to cow's milk received *Lactobacillus rhamnosus* GG (5 × 10^9^ colony-forming units) for four weeks. It was shown that the mononuclear cells of the peripheral blood from infants affected by cow's milk allergy present decreased production of interferon-γ, and that this production was increased in the group treated with probiotics. Cow's milk allergy is believed to stem from the establishment of a partial state of orally induced tolerance, and generally ceases by the age of two years, when the intestines complete their maturation. Appropriate exposure to orofecal microbes can attenuate the polarization towards Th2, which is predominant in the neonatal period, and can thereby contribute towards the emergence of a more balanced profile of cytokine production and response to food antigens. It is noteworthy that, in contrast with the beneficial effects of probiotics in early life, studies on adults have failed to show any improvement in pollen allergies following consumption of *Lactobacillus*.^33^

A further important target for therapeutic intervention is offered by polyunsaturatedfatty acids (PUFA). In 1997, Black and Sharpe reported that some recent changes in the pattern of dietary fat consumption could also play a role in the worldwide trend towards higher prevalence of allergies. In industrialized countries, as a consequence of public health measures aimed at decreasing risk factors for coronary artery disease, the consumption of saturated fats (butter and lard) has decreased and the consumption of n-6 PUFA from margarine and vegetable oils has proportionately increased. These authors suggested that these changes in diet, along with the decreased consumption of fish oils (from fresh tuna, herring, trout and salmon) or of fish oil derivatives (cod liver oil), which are rich in n-3 PUFA, eicosapentaenoic acid (EPA) and docosahexaenoic acid (DHA), contributed towards the increased prevalence of allergies.^34^

The most common PUFA species are linoleic acid (n-6) and α-linoleic acid (n-3). Linoleic acid is converted to arachidonic acid, which can be metabolized through the cyclooxygenase and lipoxygenase pathways to yield prostaglandins, thromboxanes and leukotrienes. Increased production of arachidonic acid and prostaglandin E2 favors atopic sensitization. On the other hand, α-linoleic acid inhibits metabolic conversion of linoleic acid by competing for the same enzymes.^35^ A noticeable trend in Western diets over the last decades includes decreased consumption of the anti-inflammatory n-3 PUFA, such as α-linoleic acid, and a proportional increase in the consumption of n-6 PUFA, such as linoleic acid, which can feed inflammation by providing more arachidonic acid.

Some studies involving the supplementation of adult diets with n-3 PUFA have failed to show any important benefit.^36^ However, this may be related to the fact that intervention may be ineffective once the corresponding allergic response has become established. Working within this conceptual framework, Dunstan et al.^37^ conducted a randomized controlled study on 98 pregnant women affected by atopy. These women were given either n-3 PUFAs (3.7g of n-3 PUFA with 56% as docosahexaenoic acid and 27.7% as eicosapentaenoic acid) or placebo, from the 20^th^ week of gestation to delivery. The results showed decreased neonatal cytokine response that included both Th2 and Th1 mediators (IL-5, IL-13, IL-10 and IFN-γ), thus suggesting that fish oil supplementation can have a wider immunoregulatory effect that is not limited to the Th1-Th2 polarity. Despite all this new and interesting information, the number of intervention studies using PUFA is small, and there is insufficient overall evidence to support a strong recommendation for their use as dietary supplements.

A study by Kankaanpää et al. showed that the low maternal consumption of foodstuffs rich in n-3 PUFA was associated with a higher n-6/n-3 PUFA ratio in milk, regardless of whether the mothers were atopic.^38^

Another active frontier in dietary intervention concerns the use of antioxidants, which are abundant in fresh fruits and vegetables, and accordingly lower in diets lacking these two components. Antioxidants include vitamin E, vitamin C, vitamin A, selenium and flavonoids, which are found in fresh fruit, such as apples. One of the mechanisms through which a scarcity of antioxidants in food can lead to damage is increased susceptibility of the airways to oxidative stress, thereby leading to airway inflammation and asthma.^35^ Decreased consumption of vitamin E, selenium, flavonoids and fruits by mothers during gestation, as well as by small children, may predispose to asthma. This may affect the development of the airways both through reduced antioxidant protection and through additional mechanisms such as defective modulation of Th1 cytokine production.^35^

Some studies have focused on the role of vitamin C. Soutar et al. conducted a population study that compared individuals presenting bronchopulmonary hyperreactivity, which is a hallmark of propensity to asthmatic crises, with controls. The lowest percentile of vitamin C consumption was associated with a sevenfold increase in the risk of presenting bronchopulmonary hyperreactivity.^39^ In another study, Bodner et al. conducted follow-up on individuals who developed wheezing in adulthood, in comparison with controls. The risk of developing wheezing was twice as high among those within the lowest percentile of vitamin C consumption, and five times as high for those in the lowest percentile of vitamin E consumption.^40^ Romieu et al.^41^ conducted a study that suggested that vitamin C (250 mg/ day) and vitamin E (50 mg/day) supplementation above the minimum dietary requirements among asthmatic children with low vitamin E intake provided some protection against the acute effects of ozone in their lungs.

On the other hand, selenium is an essential component of glutathione peroxidase, an enzyme that is important in oxidative metabolism. Recent studies in the UK have shown that the consumption of selenium has greatly decreased, and led to the emergence of subclinical selenium deficiency.^42^ Other studies have shown effects at the other extreme of the consumption scale: the highest percentile of vitamin E consumption was associated with the lowest risk of atopic sensitization.^43^ Another study^44^ showed that the allergen-specific proliferative response in cord blood mononuclear cells was significantly increased in subjects presenting a family history of atopic disease or of maternal tobacco consumption, but significantly decreased by a maternal diet rich in vitamin E.

According to Devereaux and Seaton, the roles played by antioxidants and PUFA in asthma are probably related and complementary.^45^

Vitamin A includes retinol, along with more than 600 carotenoid species, many of which with strong antioxidant activity.^45^ Two studies on children have shown negative associations between asthma and the serum levels of beta-carotene.^46,47^

A study by Harik-Khan et al. found that low levels of vitamin C and alpha-carotene were predictive of asthma.^48^ Another study^49^ also showed that mean serum vitamin A levels in children with asthma were significantly lower than those in controls. Interestingly, Rubin et al.^50^ found out that this relationship was influenced by exposure to cigarette smoke: in their study, a similar increase in serum beta-carotene levels was associated with a 10% reduction in asthma prevalence among nonsmokers, but with a 40% reduction in the same parameters among young people subjected to passive smoke exposure.

## CONCLUDING REMARKS

Since 1989, several studies have suggested that the fetal environment has an impact on the risk of noncommunicable diseases in adult life. This association was first described for coronary artery disease. However, it has rapidly been extended to type 2 diabetes mellitus, osteoporosis and other disturbances of metabolic and endocrine homeostasis. This has led to the paradigm of fetal origins for adult diseases, and prompted new studies that are aimed at evaluating the consequences of perinatal events on chronic diseases in adulthood.^51^

Some of the authors whose work was reviewed above have tried to define the impact of environmental influences on the fetal-maternal relationship and on the intrauterine environment, and their long-term consequences for the development of allergic disease. Changes in lifestyle, especially in the eating habits of the entire population, have been prominent over recent decades, and are likely to have had an impact on the increased prevalence of allergic disease that is detectable throughout the Western world. The hectic schedules and hassles of modern life have promoted the replacement of healthy eating habits by more practical ones that surely are not as healthy. Some studies have indicated that the use of probiotics, antioxidants, n-3 PUFA and other dietary interventions have an important effect on modulating the allergic response. However, the results among adults have not been impressive. This prompts us to focus on pregnancy, which can be seen as a period of maximum sensitivity, in which the development of tissues or systems can be permanently affected by a variety of causes, including malnutrition, hypoxia or stress.^52^ As stated by Holt et al.,^53^ the immune system immediately following birth refines a series of key functions in the presence of direct environmental stimulation, and the response patterns acquired during this critical period may persist for the entire lifespan. It is possible that this fine tuning of the immune system may already begin inside the uterus, as suggested by the evidence that the fetus can mount an immune response and is accessible to antigens and allergens.

The study by Smithells et al. using folic acid radically changed the approach towards preventing neural tube defects.^54^ Folic acid supplementation is now recommended even before conception. This shows the need for more wide-ranging intervention studies on dietary supplementation using putative immunomodulators of the various classes reviewed above, from at least the beginning of pregnancy to the first years of life, since this is the period over which the immune system matures. Only through such a comprehensive approach is it likely that the real impact from these interventions for preventing the allergic phenotypes will be defined.

## References

[1] Warner JO, Pohunet P, Marguet C, Roche WR, Clough JB (2000). Issues in understanding childhood asthma. J Allergy Clin Immunol.

[2] Beasley R, Crane J, Lai CK, Pearce N (2000). Prevalence and etiology of asthma. J Allergy Clin Immunol.

[3] Strachan DP (1989). Hay fever, hygiene, and household size. BMJ.

[4] Warner JA, Jones CA, Williams TJ, Warner JO (1998). Maternal programming in asthma and allergy. Clin Exp Allergy.

[5] Mossman TR, Cherwinski H, Bond MW, Giedlin MA, Coffman RL (1986). Two types of murine helper T cell clone. I. Definition according to profiles of lymphokine activities and secreted proteins. J Immunol.

[6] Romagnani S (1996). Th1 and Th2 in human diseases. Clin Immunol Immunopathol.

[7] Rapoport MJ, Bistritzer T, Aharoni D (2005). TH1/TH2 cytokine secretion of first degree relatives of T1DM patients. Cytokine.

[8] Legg JP, Hussain IR, Warner JA, Johnston SL, Warner JO (2003). Type 1 and type 2 cytokine imbalance in acute respiratory syncytial virus bronchiolitis. Am J Respir Crit Care Med.

[9] Wegmann TG, Lin H, Guilbert L, Mosmann TR (1993). Bidirectional cytokine interactions in the maternal-fetal relationship: is successful pregnancy a TH2 phenomenon?. Immunol Today.

[10] Arck PC, Ferrick DA, Steele-Norwood D (1999). Murine T cell determination of pregnancy outcome. Cell Immunol.

[11] Clark DA, Croitoru K (2001). TH1/TH2,3 imbalance due to cytokine-producing NK, gammadelta T and NK-gammadelta T cells in murine pregnancy decidua in success or failure of pregnancy. Am J Reprod Immunol.

[12] Warner JA, Warner JO (2000). Early life events in allergic sensitisation. Br Med Bull.

[13] Warner JA, Little SA, Pollock I, Longbottom JL, Warner JO (1991). The influence of exposure to house dust mite, cat, pollen and fungal allergens in the home on primary sensitization in asthma. Pediatr Allergy Immunol.

[14] Brown MA, Rad PY, Halonen MJ (2003). Method of birth alters interferon-gamma and interleukin-12 production by cord blood mononuclear cells. Pediatr Allergy Immunol.

[15] Liu CA, Wang CL, Chuang H, Ou CY, Hsu TY, Yang KD (2003). Prenatal prediction of infant atopy by maternal but not paternal total IgE levels. J Allergy Clin Immunol.

[16] Jones CA, Warner JA, Warner JO (1998). Fetal swallowing of IgE. Lancet.

[17] Bieber T (1997). Fc epsilon RI-expressing antigen-presenting cells: new players in the atopic game. Immunol Today.

[18] van Gool CJ, Thijs C, Dagnelie PC (2004). Determinants of neonatal IgE level: parity, maternal age, birth season and perinatal essential fatty acid status in infants of atopic mothers. Allergy.

[19] Ruiz RG, Richards D, Kemeny DM, Price JF (1991). Neonatal IgE: a poor screen for atopic disease. Clin Exp Allergy.

[20] Jones AC, Miles EA, Warner JO, Colwell BM, Bryant TN, Warner JA (1996). Fetal peripheral blood mononuclear cell proliferative responses to mitogenic and allergenic stimuli during gestation. Pediatr Allergy Immunol.

[21] Daffos F, Forestier F, Grangeot-Keros L (1984). Prenatal diagnosis of congenital rubella. Lancet.

[22] Gill TJ, Repetti CF, Metlay LA (1983). Transplacental immunization of the human fetus to tetanus by immunization of the mother. J Clin Invest.

[23] Vance GH, Holloway JA (2002). Early life exposure to dietary and inhalant allergens. Pediatr Allergy Immunol.

[24] Viscarello RR, Cullen MT, DeGennaro NJ, Hobbins JC (1992). Fetal blood sampling in human immunodeficiency virus--seropositive women before elective midtrimester termination of pregnancy. Am J Obstetr Gynecol.

[25] Kilshaw PJ, Cant AJ (1984). The passage of maternal dietary proteins into human breast milk. Int Arch Allergy Appl Immunol.

[26] Szepfalusi Z, Pichler J, Elsasser S (2000). Transplacental priming of the human immune system with environmental allergens can occur early in gestation. J Allergy Clin Immunol.

[27] Spencer J, MacDonald TT, Finn T, Isaacson PG (1986). The development of gut associated lymphoid tissue in the terminal ileum of fetal human intestine. Clin Exp Immunol.

[28] Butland BK, Strachan DP, Anderson HR (1999). Fresh fruit intake and asthma symptoms in young British adults: confounding of effect modification by smoking?. Eur Respir J.

[29] Langley-Evans S (1997). Fetal programming of immune function and respiratory disease. Clin Exp Allergy.

[30] Kalliomaki M, Salminen S, Arvilommi H, Kero P, Koskinen P, Isolauri E (2001). Probiotics in primary prevention of atopic disease: a randomised placebo-controlled trial. Lancet.

[31] Vaarala O (2003). Immunological effects of probiotics with special reference to lactobacilli. Clin Exp Allergy.

[32] Pohjavuori E, Viljanen M, Korpela R (2004). Lactobacillus GG effect in increasing IFN-gamma production in infants with cow's milk allergy. J Allergy Clin Immunol.

[33] Helin T, Haahtela S, Haahtela T (2002). No effect of oral treatment with an intestinal bacterial strain, Lactobacillus rhamnosus (ATCC 53103), on birch-pollen allergy: a placebo-controlled double-blind study. Allergy.

[34] Black PN, Sharpe S (1997). Dietary fat and asthma: is there a connection?. Eur Respir J.

[35] Devereux G, Seaton A (2005). Diet as a risk factor for atopy and asthma. J Allergy Clin Immunol.

[36] Woods RK, Thien FC, Abramson MJ (2000). Dietary marine fatty acids (fish oil) for asthma. Cochrane Database Syst Rev.

[37] Dunstan JA, Mori TA, Barden A (2003). Fish oil supplementation in pregnancy modifies neonatal allergen-specific immune responses and clinical outcomes in infants at high risk of atopy: a randomized, controlled trial. J Allergy Clin Immunol.

[38] Kankaanpaa P, Nurmela K, Erkkila A (2001). Polyunsaturated fatty acids in maternal diet, breast milk, and serum lipid fatty acids of infants in relation to atopy. Allergy.

[39] Soutar A, Seaton A, Brown K (1997). Bronchial reactivity and dietary antioxidants. Thorax.

[40] Bodner C, Godden D, Brown K, Little J, Ross S, Seaton A (1999). Antioxidant intake and adult-onset wheeze: a case-control study. Aberdeen WHEASE Study Group. Eur Respir J.

[41] Romieu I, Sienra-Monge JJ, Ramírez-Aguilar M (2002). Antioxidant supplementation and lung functions among children with asthma exposed to high levels of air pollutants. Am J Respir Crit Care Med.

[42] Brown KM, Pickard K, Nicol F, Arthur JR (1997). Selenium status and selenoprotein function in a Scottish population. Proc Nutr Soc.

[43] Fogarty A, Lewis S, Weiss S, Britton J (2000). Dietary vitamin E, IgE concentrations, and atopy. Lancet.

[44] Devereux G, Barker RN, Seaton A (2002). Antenatal determinants of neonatal immune responses to allergens. Clin Exp Allergy.

[45] Rubin RN, Navon L, Cassano PA (2004). Relationship of serum antioxidants to asthma prevalence in youth. Am J Respir Crit Care Med.

[46] Harik-Khan RI, Muller DC, Wise RA (2004). Serum vitamin levels and the risk of asthma in children. Am J Epidemiol.

[47] Seaton A, Devereux G (2000). Diet, infection and wheezy illness: lessons from adults. Pediatr Allergy Immunol.

[48] Harik-Khan RI, Muller DC, Wise RA (2004). Serum vitamin levels and the risk of asthma in children. Am J Epidemiol.

[49] Arora P, Kumar V, Batra S (2002). Vitamin A status in children with asthma. Pediatr Allergy Immunol.

[50] Rubin RN, Navon L, Cassano PA (2004). Relationship of serum antioxidants to asthma prevalence in youth. Am J Respir Crit Care Med.

[51] Hanson M, Gluckman P, Bier D (2004). Report on the 2nd World Congress on Fetal Origins of Adult Disease, Brighton, U.K., June 7-10, 2003. Pediatr Res.

[52] Barker DJ (2004). Developmental origins of adult health and disease. J Epidemiol Community Health.

[53] Holt PG, Jones CA (2000). The development of the immune system during pregnancy and early life. Allergy.

[54] Botto LD, Lisi A, Robert-Gnansia E (2005). International retrospective cohort study of neural tube defects in relation to folic acid recommendations: are the recommendations working?. BMJ.

